# *In Silico* Analysis of Functional Single Nucleotide Polymorphisms in the Human *TRIM22* Gene

**DOI:** 10.1371/journal.pone.0101436

**Published:** 2014-07-01

**Authors:** Jenna N. Kelly, Stephen D. Barr

**Affiliations:** Western University, Schulich School of Medicine and Dentistry, Center for Human Immunology, Department of Microbiology and Immunology, Dental Sciences Building, London, Ontario, Canada; University of South Florida College of Medicine, United States of America

## Abstract

Tripartite motif protein 22 (TRIM22) is an evolutionarily ancient protein that plays an integral role in the host innate immune response to viruses. The antiviral TRIM22 protein has been shown to inhibit the replication of a number of viruses, including HIV-1, hepatitis B, and influenza A. TRIM22 expression has also been associated with multiple sclerosis, cancer, and autoimmune disease. In this study, multiple *in silico* computational methods were used to identify non-synonymous or amino acid-changing SNPs (nsSNP) that are deleterious to TRIM22 structure and/or function. A sequence homology-based approach was adopted for screening nsSNPs in TRIM22, including six different *in silico* prediction algorithms and evolutionary conservation data from the ConSurf web server. In total, 14 high-risk nsSNPs were identified in TRIM22, most of which are located in a protein interaction module called the B30.2 domain. Additionally, 9 of the top high-risk nsSNPs altered the putative structure of TRIM22's B30.2 domain, particularly in the surface-exposed v2 and v3 regions. These same regions are critical for retroviral restriction by the closely-related TRIM5α protein. A number of putative structural and functional residues, including several sites that undergo post-translational modification, were also identified in TRIM22. This study is the first extensive *in silico* analysis of the highly polymorphic *TRIM22* gene and will be a valuable resource for future targeted mechanistic and population-based studies.

## Introduction

Single nucleotide polymorphisms (SNPs), defined as single base changes in a DNA sequence, are responsible for the majority of genetic variation in the human population. Although many SNPs are phenotypically neutral, non-synonymous SNPs (nsSNPs) often have deleterious effects on protein structure or function. NsSNPs are located in protein coding regions and result in an amino acid substitution in the corresponding protein product. As such, nsSNPs can alter the structure, stability, or function of proteins, and are often associated with human disease. Indeed, previous studies have shown that approximately 50% of the mutations involved in inherited genetic disorders are due to nsSNPs [1]–[3]. Recently, a number of genetic studies have focused on nsSNPs in innate immune genes. These studies have identified multiple nsSNPs that influence susceptibility to infection, as well as the development of inflammatory disorders and autoimmune diseases [4]–[9]. Nonetheless, because innate immune genes are often highly polymorphic, many nsSNPs in these genes remain uncharacterized.

Members of the tripartite motif (TRIM) protein family are involved in a wide range of biological processes related to innate immunity [10]–[12]. TRIM proteins are defined by an RBCC motif, which consists of a RING domain, one or two B-box domains, and a predicted coiled-coil region. Most TRIM proteins also have a protein interaction module called a B30.2 domain at their C-terminus [13]–[15]. Many TRIM proteins are induced by interferon signaling and several possess antiviral activity, in particular against the *Retroviridae* family of viruses. Recent studies have implicated TRIM proteins in the regulation of pathogen-recognition signaling pathways, a finding that has sparked considerable interest in understanding how TRIM family proteins contribute to the innate immune response [16]–[21].

One well-studied member of the TRIM family, TRIM5α, is required for the species-specific block against HIV-1 replication in primate cells [22]–[24]. Recently, TRIM5α was also shown to promote innate immune signaling and to function as an innate immune sensor for the retrovirus capsid lattice *in vitro*. Previous studies have established that TRIM5α binds to the HIV-1 capsid protein in the mature viral core via four variable regions (v1-v4) in its B30.2 domain [25], [26]. The v1 or ‘antiviral patch’ region was previously shown to be the major determinant for species-specific HIV-1 restriction by TRIM5α. Mutations in the other variable regions (v2-v4) have also been shown to interfere with TRIM5α-mediated restriction of HIV-1, SIV, and N-MLV [22], [26]–[29]. Notably, analogous variable regions are found in several other B30.2-containing TRIM proteins [30], [31], [32].

Human *TRIM5* is located on chromosome 11 within a cluster of four closely-related *TRIM* genes that also includes *TRIM6*, *TRIM22*, and *TRIM34*. *TRIM5* and *TRIM22* have an ancient and dynamic evolutionary relationship, whereby both genes have evolved under positive selection for millions of years in a mutually exclusive manner [33]. Similar to TRIM5α, TRIM22 has also been shown to inhibit HIV-1 replication in a number of human cell lines and primary monocyte-derived macrophages [34]–[37]. TRIM22 expression levels have also been shown to influence HIV-1 infection *in vivo* [37], [38], [39]. Interestingly, nsSNPs in TRIM5α, including H43Y, R136Q, and G249D, significantly alter HIV-1 acquisition and disease progression in humans [40]–[43]. Despite TRIM22's highly polymorphic nature, it is unknown how nsSNPs affect its biological and/or antiviral functions. Here, multiple *in silico* computational methods were used to identify nsSNPs in the *TRIM22* gene that are predicted to be highly deleterious to TRIM22 structure and/or function. A total of 14 high-risk nsSNPs were identified, including 9 that altered the putative structure of TRIM22's B30.2 domain. A number of sites predicted to undergo post-translational modification (ubiquitylation, sumoylation, phosphorylation) were also identified. This study is the first extensive *in silico* analysis of the *TRIM22* gene and will establish a strong foundation for future structure-function and population-based studies.

## Materials and Methods

### Retrieval of SNPs

Polymorphism data for the *TRIM22* gene were retrieved from the following databases: the UniProt database (http://www.uniprot.org) (UniProtKB ID Q8IYM9), the NCBI dbSNP database (https://www.ncbi.nlm.nih.gov/SNP/), 1000 Genomes (http://www.1000genomes.org/), and the Ensembl genome browser (http://www.ensembl.org/index.html). Minor allele frequencies were obtained from the NCBI dbSNP database, the Ensembl genome browser, and the 1000 Genomes browser [44]–[46].

### Non-synonymous SNP analysis

Functional effects of nsSNPs were predicted using the following *in silico* algorithms: Polyphen-2 (http://genetics.bwh.harvard.edu/pp2), SIFT (http://sift.jcvi.org/), nsSNP Analyzer (http://snpanalyzer.uthsc.edu/), PhD-SNP (http://snps.biofold.org/phd-snp/phd-snp.html), SNPs&GO (http://snps-and-go.biocomp.unibo.it/snps-and-go/), and PMut (mmb2.pcb.ub.es:8080/PMut) [47]–[52]. nsSNPs predicted to be deleterious by at least four *in silico* algorithms were categorized as high-risk nsSNPs and were selected for further analysis.

### Phylogenetic analysis

Evolutionary conservation of amino acid residues in TRIM22 was determined using the ConSurf web server (consurf.tau.ac.il/) [53]. In ConSurf, 14 TRIM22 homologues were aligned and position-specific conservation scores were calculated using an empirical Bayesian algorithm (Conservation Scores: 1–4 Variable, 5–6 Intermediate, and 7–9 Conserved). Putative functional and structural residues were also predicted using ConSurf by combining evolutionary conservation scores with solvent accessibility predictions (Figures S1 and S2). Highly conserved amino acids that were located at high-risk nsSNP sites were selected for further analysis.

### Structural analysis

3D-Jigsaw was used to generate 3D structural models for wild type TRIM22 (UniProtKB Q8IYM9) and each of the 9 high-risk nsSNPs in TRIM22's B30.2 domain. For each model, only the B30.2 sequence was submitted. 3D-Jigsaw searches multiple sequence databases (e.g. PFAM and PDB) and builds structures based on homologues of known structure [54]. Models were viewed using the Swiss-PdbViewer (http://www.expasy.org/spdbv/) [55]. Tm-Align was used to calculate Tm-scores and root mean square deviation (RMSD) (http://zhanglab.ccmb.med.umich.edu/TM-align/) [56].

### Prediction of post-translational modification sites

Putative ubiquitylation sites were predicted using the UbPred (www.ubpred.org) and BDM-PUB (bdmpub.biocuckoo.org) programs [2]. In UbPred, lysine residues with a score of ≥0.62 were considered ubiquitylated. For BDM-PUB, the balanced cut-off option was selected. Putative sumoylation sites were predicted using the SUMOplot (http://www.abgent.com/sumoplot) and SUMOsp 2.0 (http://sumosp.biocuckoo.org/) programs [57]. For SUMOplot, only high probability motifs with a score >0.5 were considered sumoylated. Medium level threshold with a 2.64 cut-off value was selected for SUMOsp 2.0 analysis. Putative phosphorylation sites were predicted using GPS 2.1 (http://gps.biocuckoo.org/) and NetPhos 2.0 (http://www.cbs.dtu.dk/services/NetPhos/) [58], [59]. For GPS 2.1 analysis, high level threshold with cut-off values ranging from 0.776-11 were selected. In NetPhos 2.0, serine, threonine, and tyrosine residues with a score of >0.5 were considered phosphorylated. Sumo-interacting motifs (SIM) were identified manually and compared to experimentally verified SIMs in the scientific literature [60], [61].

### Protein stability analysis

I-Mutant version 2.0, an online support vector machine tool based on the ProTherm database, was used to evaluate nsSNP-induced changes in protein stability [62]. nsSNP protein-coding sequences were submitted to I-Mutant 2.0 for 2 high-risk nsSNPs that coincide with putative PTM sites, 5 low-risk nsSNPs that coincide with putative PTM sites, and 12 additional high-risk nsSNPs that do not coincide with predicted PTM sites. I-Mutant 2.0 estimates the free energy change value (DDG) by calculating the unfolding Gibbs free energy value (ΔG) for the wild type protein and subtracting it from that of the mutant protein (DDG or ΔΔG  =  ΔG mutant – ΔG wild type). It also predicts the sign (increase or decrease) of the free energy change value (DDG), along with a reliability index for the results (RI: 0–10, where 0 is the lowest reliability and 10 is the highest reliability). A DDG <0 corresponds to a decrease in protein stability, whereas a DDG >0 corresponds to an increase in protein stability. However, according to the ternary classification system (SVM3), a large decrease in protein stability corresponds to a DDG <−0.5 and a large increase in protein stability corresponds to a DDG >0.5. In contrast, DDG values that fall between −0.5 and 0.5 correspond to relatively neutral protein stability [62], [63]. The pH was set to 7 and the temperature was set to 25°C for all submissions.

## Results and Discussion

### SNP dataset

Polymorphism data for the *TRIM22* gene were retrieved from the NCBI dbSNP database, the Ensembl genome browser, and the UniProt database [44]–[46]. According to these databases, the *TRIM22* gene contains 66 nsSNPs, 8 SNPs in its 5′ UTR, and 32 SNPs in its 3′ UTR. Of the 66 nsSNPs, 10 generate truncated versions of the TRIM22 protein (nonsense and frameshift mutations), whereas 56 introduce single amino acid changes (missense mutations) into TRIM22 (Table S1). To determine whether a given missense mutation affected TRIM22 function, we subjected the latter 56 nsSNPs to a variety of *in silico* SNP prediction algorithms. The results, which are summarized in Table 1, identified a number of nsSNPs with a high probability of being deleterious to TRIM22 structure and/or function.

**Table 1:** Summary of prediction results for nsSNPs in the TRIM22 protein.

Prediction	Number of nsSNPs (%)					
	PP-2	SIFT	nsSNP AZ	PhD-SNP	PMUT	SNPs&GO
Deleterious	13 (23)	19 (34)	-	-	-	-
PD	10 (18)	-	-	-	-	-
Benign	33 (59)	37 (66)	-	-	-	-
Disease	-	-	21 (38)	25 (45)	25 (45)	11 (20)
Neutral	-	-	35 (62)	31 (55)	31 (55)	45 (80)

Percentage of total nsSNPs (56) shown in parentheses for each category; PD: possibly deleterious; PP-2: Polyphen-2; nsSNP AZ: nsSNP Analyzer.

### Non-synonymous SNP analysis

Our analyses included the following six *in silico* SNP prediction algorithms: Polyphen-2, SIFT, nsSNP Analyzer, PhD-SNP, PMUT, and SNPs&GO [47]–[52]. According to our Polyphen-2 results, 13 nsSNPs (23%) are damaging to TRIM22 function, whereas 33 nsSNPs (59%) are benign. An additional 10 nsSNPs (18%) are predicted to be ‘possibly damaging’ by Polyphen-2 (Table 1). Our SIFT analysis predicted that 19 nsSNPs (34%) are deleterious to TRIM22 function and 37 nsSNPs (66%) are tolerated. On the contrary, the nsSNP Analyzer predicted that 21 nsSNPs (38%) cause disease and 35 nsSNPs (62%) are neutral (Table 1). Both PhD-SNP and PMUT predicted that 25 (45%) nsSNPs are pathological and 31 (55%) nsSNPs are neutral (Table 1). SNPs&GO analysis, which includes information from the Gene Ontology annotation, predicted that 11 nsSNPs (20%) cause disease and 45 nsSNPs (80%) are neutral (Table 1). Interestingly, we found that the majority of potentially deleterious nsSNPs were located in the B30.2 domain, including 3 nsSNPs that were predicted to be damaging by all six SNP prediction algorithms (P403T, T460I, and C494F). Because each algorithm uses different parameters to evaluate the nsSNPs, nsSNPs with more positive results are more likely to be truly deleterious. Here, we classified nsSNPs as high-risk if they were predicted to be deleterious by four or more SNP prediction algorithms. 14 nsSNPs met this criteria and were selected for further analysis (Table 2, see Table S2 for all 56 nsSNP prediction results).

**Table 2:** TRIM22 nsSNPs predicted to be functionally significant by four or more SNP prediction algorithms.

nsSNP ID	Mutation	Domain	# Del. Pred.
rs201847190	L68R	Spacer 1	5
rs199625192	H73R	Spacer 1	5
rs368058642	E135K	Coiled-coil	4.5
rs374292901	I234N	Spacer 2	5
rs61735273	S244L	Spacer 2	5
rs371728648	G346S	B30.2	5
rs191847788	K364N	B30.2	4.5
rs375595000	P403T	B30.2	6
rs370495523	L432W	B30.2	4
rs187416296	R442C	B30.2	5
rs377529439	F456I	B30.2	5
rs371028900	T460I	B30.2	6
rs200638791	P484S	B30.2	4.5
rs200148337	C494F	B30.2	6

# Del. Pred.  =  number of deleterious predictions.

### Conservation profile of high-risk non-synonymous SNPs

Amino acids that are involved in important biological processes, such as those located in enzymatic sites or required for protein-protein interactions, tend to be more conserved than other residues. As such, nsSNPs that are located at highly conserved amino acid positions tend to be more deleterious than nsSNPs that are located at non-conversed sites [3], [64]. To further investigate the potential effects of the 14 high-risk nsSNPs in Table 2, we calculated the degree of evolutionary conservation at all amino acid sites in the TRIM22 protein using the ConSurf web server. ConSurf employs an empirical Bayesian method to determine evolutionary conservation and identify putative structural and functional residues [53]. For the purpose of this study, we focused on amino acid sites that coincide in location with the 14 high-risk nsSNPs; however, ConSurf also identified a number of other residues that may be functionally relevant (Figures S1 and S2).

ConSurf analysis revealed that residues L68, H73, E135, I234, S244, G346, K364, P403, L432, R442, F456, T460, and C494 are highly conserved (Conservation Score of 7–9). In addition, ConSurf predicted that T460 was an important structural residue (highly conserved and buried) and that L68, K364, and P403 were important functional residues (highly conserved and exposed) (Table 3). To identify putative structural and functional sites, ConSurf combines evolutionary conservation data with solvent accessibility predictions. Highly conserved residues are predicted to be either structural or functional based on their location relative to the protein surface or protein core [65]. Remarkably, two of the three high-risk nsSNPs that were predicted to be deleterious by all six SNP prediction algorithms (P403T and T460I) were also identified as important structural or functional residues by ConSurf (Table 2, 3). Taken together, our data strongly suggest that the nsSNPs P403T and T460I are deleterious to TRIM22 structure and/or function.

**Table 3:** Conservation profile of amino acids in TRIM22 that coincide in location with high-risk nsSNPs.

nsSNP ID	Amino Acid	CS	ConSurf prediction
rs201847190	L68	8	Highly conserved and exposed (f)
rs199625192	H73	7	Exposed
rs368058642	E135	7	Exposed
rs374292901	I234	7	Buried
rs61735273	S244	8	Buried
rs371728648	G346	8	Buried
rs191847788	K364	9	Highly conserved and exposed (f)
rs375595000	P403	8	Highly conserved and exposed (f)
rs370495523	L432	8	Buried
rs187416296	R442	7	Exposed
rs377529439	F456	8	Buried
rs371028900	T460	9	Highly conserved and buried (s)
rs200638791	P484	6	Exposed
rs200148337	C494	8	Buried

CS: conservation score (1–4 =  variable, 5 =  average, 6–9 =  conserved); (f): predicted functional site, (s): predicted structural site.

### Comparative modeling of high-risk non-synonymous SNPs

**Figure 1:**
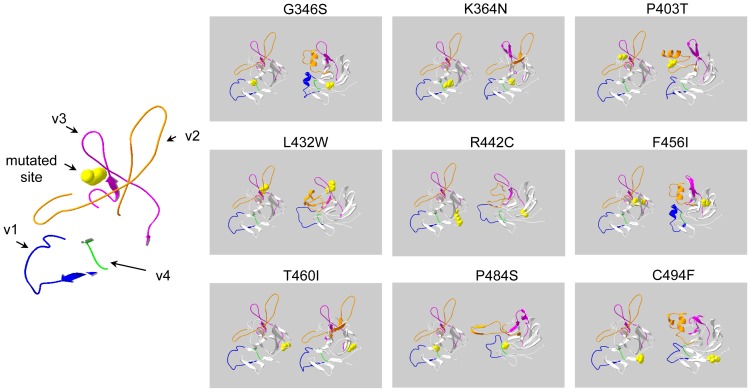
Structural models for wild type TRIM22 and high-risk nsSNPs in the B30.2 domain. Putative structural models for the B30.2 domains of wild type TRIM22 and the 9 high-risk nsSNPs located in the B30.2 domain. Variable regions (v1-v4) are highlighted as follows: v1 blue, v2 orange, v3 magenta, and v4 green. Non-variable regions are shown in white and mutated amino acids are shown in yellow. Left image: Enlarged reference image that illustrates the color and location of each variable region and the color of mutated amino acids (image shown is the v1-v4 regions of wild type TRIM22 and the P403 amino acid). Each of the 9 nsSNP images (small images on the right) show the putative 3D structure of wild type TRIM22's B30.2 domain on the left and the putative 3D structure of TRIM22's B30.2 domain with the mutated amino acid (nsSNP) on the right. The location of the amino acid in question is shown (yellow) on both wild type and nsSNP structures. All models were generated using the 3D-JigSaw protein comparative modeling server and SPDBV (v4.1).

To examine whether P403T and T460I altered the 3D structure of TRIM22's B30.2 domain, we individually substituted each nsSNP into the wild type TRIM22 sequence and submitted the sequences to 3D-Jigsaw for structural analysis. We also submitted sequences for the remaining 7 high-risk nsSNPs in the B30.2 domain (i.e. G346S, K364N, L432W, R442C, F456I, P484S, and C494F) since our *in silico* and ConSurf results indicated that these nsSNPs were also highly likely to be deleterious. Theoretical structural models were generated for each nsSNP using the 3D-Jigsaw program, which constructs 3D models for proteins based on homologues of known structure [54]. We then used Swiss-PdbViewer to compare each nsSNP model to the predicted 3D-Jigsaw model of wild type TRIM22 [55]. All of the nsSNPs altered the putative 3D structure of wild type TRIM22's B30.2 domain. G346S, P40T, L432W, F456I, and C494F introduced an alpha helix into the v2 region, whereas the other 4 nsSNPs introduced beta strands into the v2 region (Figure 1). With the exception of P484S, which introduced an alpha helix into the v3 region, all of the nsSNP models contained elongated and/or additional beta strands in the v3 region. Only G346S and F456I altered the v1 region (both introduced an alpha helix); however, all 9 nsSNPs altered the length and/or number of beta strands in non-variable regions of the B30.2 domain. Notably, P484S was the only nsSNP model that contained fewer beta strands than wild type TRIM22 in certain regions (Figure 1). The majority of nsSNP models contained a greater number of beta strands than wild type TRIM22, resulting in overall net increase in beta strand formation.

To extend our structural analysis, we used Tm-Align to calculate the Tm-score and root mean square deviation (RMSD) for each nsSNP model. Tm-score is used to assess topological similarity between wild type and mutant models, whereas RMSD is used to measure average distance between the α-carbon backbones of wild type and mutant models [56], [66]. A higher RMSD typically indicates greater deviation between wild type and mutant structures. The Tm-score and RMSD for each nsSNP model is listed in Table 4. The maximum RMSD was 3.04 (R442C), followed by 3.03 (F456I), 3.00 (L432W), 2.96 (G346S), and 2.80 (P484S). RMSD for nsSNPs K364N, P403T, T460I, and C494F ranged from 1.58 to 1.99 Å. These results indicate that 9 high-risk nsSNPs markedly alter the putative structure of TRIM22's B30.2 domain, in particular the surface-exposed v2 and v3 regions, and that they likely induce severe structural changes in the TRIM22 protein.

**Table 4:** RMSD (Å) and TM-score for the 9 high-risk nsSNPs in the B30.2 domain of TRIM22.

nsSNP ID	Mutation	RMSD (Å)	TM-Score
rs371728648	G346S	2.96	0.75184
rs191847788	K364N	1.72	0.93911
rs375595000	P403T	1.99	0.85389
rs370495523	L432W	3.00	0.70821
rs187416296	R442C	3.04	0.68305
rs377529439	F456I	3.03	0.73743
rs371028900	T460I	1.76	0.94873
rs200638791	P484S	2.80	0.75981
rs200148337	C494F	1.58	0.95645

RMSD and Tm-scores were calculated using Tm-Align.

Importantly, these nsSNPs may decrease flexibility in the v2 and v3 regions of TRIM22. The v2/v3 regions of wild type TRIM22 are predicted to form relaxed loop segments, similar to the loops in the recently solved 3D structure of rhesus monkey TRIM5α's B30.2 domain [26]. In contrast, the v2 and v3 regions of the nsSNP models contain more rigid secondary structures, such as alpha helices or beta strands (Figure 1). Since loop flexibility in rhesus monkey TRIM5α is thought to facilitate restriction of divergent retroviruses and to increase resistance to mutations in the HIV-1 capsid protein, it is possible that these nsSNPs may impair the antiviral activity and/or breadth of TRIM22. Further experiments, such as the resolution of wild type TRIM22's tertiary structure, are required to address these possibilities.

### Prediction of post-translational modification sites in TRIM22

To investigate how nsSNPs may influence the post-translational modification (PTM) of TRIM22, we used a variety of *in silico* prediction tools to identify putative PTM sites in the TRIM22 protein. PTMs are involved in many biological processes, including a number of canonical innate immune pathways, and are essential for the regulation of protein structure and function [57], [67]–[69]. To analyze residues in TRIM22 that may undergo ubiquitylation or sumoylation, we used the UbPred, BDM-PUB, SUMO-plot, and SUMOsp 2.0 programs. The GPS 2.1 and NetPhos 2.0 servers were used to predict serine, threonine, and tyrosine phosphorylation sites in the TRIM22 protein [2], [58], [59], [70].

UbPred predicted that 6 lysine residues in TRIM22 undergo ubiquitylation. In contrast, BDM-PUB predicted that 19 lysine residues undergo ubiquitylation. Both UbPred and BDM-PUB predicted that residues K63, K160, and K173 undergo ubiquitylation (Table 5). According to ConSurf, these 3 lysine residues are highly conserved and exposed to the protein surface. ConSurf also predicted that K173 was a functional residue (Figure S1). SUMOplot predicted that 4 lysine residues in TRIM22 undergo sumoylation, whereas SUMOsp 2.0 predicted that 2 lysine residues undergo sumoylation. Both programs predicted that K153 undergoes sumoylation (Table 5). Similar to K173, ConSurf showed that K153 is highly conserved and exposed to the protein surface. ConSurf also predicted that K153 was a functional residue (Figure S1).

**Table 5:** Putative ubiquitylation and sumoylation sites in the TRIM22 protein.

Ubiquitylation		Sumoylation	
UbPred	BDM-PUB	SUMOplot	SUMOsp 2.0
93 (7e)*	6 (3e)	6 (3e)	85 (2e)
160 (7e)*	44 (1e)	153 (9e)*	153 (9e)*
173 (9e)*	85 (2e)	185 (4e)	
204 (6e)	93 (7e)*	265 (6e)	
257 (1e)	103 (6e)		
430 (6e)	**109 (9e)**		
	160 (7e)*		
	173 (9e)*		
	265 (6e)		
	**266 (9e)**		
	268 (2e)		
	272 (6e)		
	**273 (9e)**		
	275 (7e)		
	324 (1e)		
	332 (2e)#		
	374 (1e)		
	380 (3e)		
	382 (1e)		

Conservation score (CS) shown in parentheses (see Table 3 and Figure S1) following amino acid site; Putative functional residues are indicated with bold text, whereas putative structural residues are indicated with italicized text (Figure S1); Residues predicted to undergo ubiquitylation or sumoylation by both programs are indicated with an asterisk; Residues predicted to undergo ubiquitylation or sumoylation that coincide with the location of nsSNPs are indicated with a hashtag.

**Figure 2:**
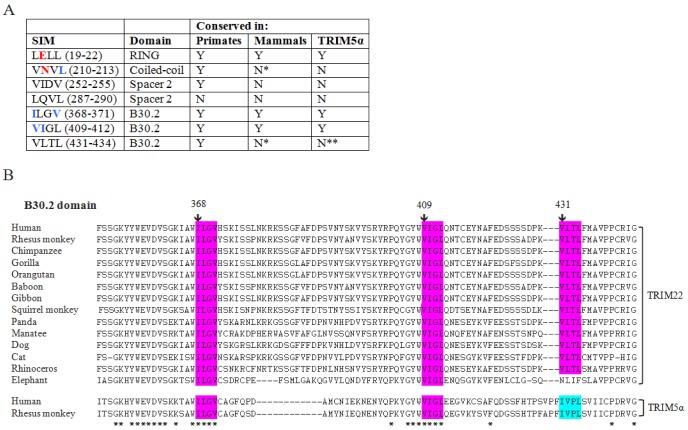
Putative sumo-interacting motifs (SIMs) in TRIM22. **A**. List of putative SIMs in the TRIM22 protein, including the sequence and domain location for each SIM (amino acids are indicated in parentheses); Red and blue amino acids are predicted functional and structural residues, respectively (ConSurf analysis Figure S1); Asterisk: SIMs that are conserved in all mammalian TRIM22 orthologues except elephant; Double asterisk: SIMs that are not found in TRIM5α, but are replaced by a different SIM (e.g. VLTL, IVPL). **B**. Alignment of mammalian TRIM22, human TRIM5α, and rhesus monkey TRIM5α amino acid sequences (amino acids 350–444 of the B30.2 domain are shown). Conserved SIMs are highlighted in magenta and other SIMs are highlighted in light blue. Conserved amino acids are indicated with an asterisk.

In addition to putative sumoylation sites, we also identified 7 potential sumo-interacting motifs (SIM) (Figure 2A). SIMs are short hydrophobic motifs that interact non-covalently with other sumoylated proteins. The best characterized SIMs have the consensus sequence V/I/L-x-V/I/L-V/I/L or V/I/L-V/I/L-x-V/I/L [61]. Notably, 5 of the putative SIMs are highly conserved in multiple TRIM22 orthologues and 3 are also present in the human and rhesus monkey TRIM5α proteins (Figure 2B). In addition, 2 TRIM5α SIMs (ILGV and VIGL) were previously shown to be required for TRIM5α-mediated antiviral activity. SIM mutations in the rhesus monkey TRIM5α protein abolished HIV-1 restriction and disrupted TRIM5α trafficking to SUMO-1 nuclear bodies. Moreover, SIM mutations in the human TRIM5α protein abrogated N-MLV restriction by preventing TRIM5α binding to the sumoylated N-MLV capsid protein [60], [71]. More studies are needed to determine the role that SIMs play in TRIM22-mediated antiviral activity.

To identify putative phosphorylation sites in TRIM22, we used GPS 2.1 and NetPhos 2.0 servers. The GPS 2.1 server predicted that there were 31 serine-specific phosphorylation sites, 13 threonine-specific sites, and 11 tyrosine-specific sites in the TRIM22 protein. Conversely, NetPhos 2.0 predicted that there were 19 serine-specific phosphorylation sites, 4 threonine-specific sites, and 2 tyrosine-specific sites (Table 6). 16 serine residues, 3 threonine residues, and 2 tyrosine residues were predicted to be phosphorylated by both GPS 2.1 and NetPhos 2.0 servers. Many of these putative phosphorylation sites are highly conserved among multiple TRIM22 orthologues and several were predicted to be important structural or functional residues by ConSurf (Table 6, Figure S1). Although TRIM22 phosphorylation has never been demonstrated experimentally, our results suggest that it may undergo phosphorylation at a number of sites. Of interest, other TRIM proteins have been shown to undergo phosphorylation, including the antiviral TRIM19 and TRIM21 proteins [72]–[76].

**Table 6:** Putative phosphorylation sites in the TRIM22 protein.

GPS 2.1			NetPhos 2.0		
Serine	Threonine	Tyrosine	Serine	Threonine	Tyrosine
4 (1e)	23 (7e)	175 (1b)	46 (7e)*	130 (7b)	356 (8b)*
**27 (9e)**	61 (1b)#	298 (1e)	50 (1e)	263 (3e)*	479 (5b)*
46 (7e)*	170 (1e)	299 (6b)	54 (3e)*	325 (1e)*	
54 (3e)*	220 (1e)	355 (5b)	87 (4e)*	330 (1e)*#	
87 (4e)*	232 (1e)#	356 (8b)*	244 (8b)*#		
**122 (9e)**	263 (3e)*	394 (1b)	245 (8b)*		
231 (4e)	294 (7e)#	398 (7b)	259 (9e)*		
**235 (9e)**	311 (2b)	418 (8b)	261 (2e)*		
244 (8b)*#	325 (1e)*	467 (8b)	269 (1e)*		
245 (8b)*	330 (1e)*#	479 (5b)*	271 (8e)*		
259 (9e)*	433 (7b)	481 (8e)*	276 (5e)*		
261 (2e)*	*460 (9b)#*		284 (5e)*		
269 (1e)*	492 (6e)		373 (8b)*		
271 (8e)*			383 (3e)*		
276 (5e)*			384 (9e)*		
284 (5e)*			399 (7b)		
**309 (8e)**			425 (6e)*		
312 (6e)			426 (4e)*		
*317 (9b)*			**475 (8e)**		
373 (8b)*					
376 (2e)*					
377 (1e)					
383 (3e)*					
384 (9e)*					
391 (3e)					
424 (7e)					
425 (6e)*					
426 (4e)*					
*455 (9b)*					
**497 (9e)**					
498 (7e)					

Conservation score (CS) shown in parentheses (see Table 3 and Figure S1) following amino acid site; Putative functional residues are indicated with bold text, whereas putative structural residues are indicated with italicized text (Figure S1); Residues predicted to undergo phosphorylation by both GPS 2.1 and NetPhos 2.0 are indicated with an asterisk; Residues predicted to undergo phosphorylation that also coincide with the location of nsSNPs are indicated with a hashtag.

Several putative PTMs coincide in location with nsSNPs in the *TRIM22* gene (T61, T232, S244, T294, T330, K332, and T460). S244 and T460 are particularly interesting because both sites are highly conserved among TRIM22 orthologues and S244L and T460I were predicted to be deleterious by 5 and 6 *in silico* algorithms, respectively (Table 2, 3). In addition, T460 was predicted to be a critical structural residue by ConSurf. Although the consequences of TRIM22 phosphorylation are currently unknown, the mutation of phosphorylation sites in other proteins has been shown to profoundly alter protein function by, for example, altering protein stability, localization, or protein-protein interactions. To this end, we used I-Mutant to predict whether S244L and T460I altered the stability of the TRIM22 protein. I-Mutant is a support vector machine-based tool that predicts changes in protein stability following single site mutations by estimating free energy changes as well as the direction of the change (increase or decrease) [62]. Both S244L and T460I were predicted to be less stable than the wild type protein, with free energy change values of −0.83 and −1.38, respectively (Table 7). The I-Mutant results for the 12 high-risk nsSNPs that do not coincide with putative PTM sites, plus the results for the 5 low-risk nsSNPs that do coincide with putative PTM sites, are also shown in Table 7.

**Table 7:** I-Mutant results for selected nsSNPs in the TRIM22 protein.

nsSNP ID	Mutation	# Del. Pred.	DDG	Sign of DDG	PTM	ConSurf
rs192306924	T61N	1	0.56	Decrease (1)	Yes	1b
**rs201847190**	**L68R**	**5**	**−1.02**	Decrease (7)*	**No**	**8e**
rs199625192	H73R	5	0.23	Decrease (3)	No	7e
rs368058642	E135K	4.5	−1.00	Decrease (9)*	No	7e (9b)
rs2291843	T232A	0	−0.53	Decrease (5)	Yes	1e
rs374292901	I234N	5	−0.80	Decrease (1)	No	7b (9e)
rs61735273	S244L	5	−0.83	Decrease (2)	Yes	8b
rs73404240	T294K	2	−0.63	Decrease (5)	Yes	7e
rs201494620	T330I	1	−2.14	Decrease (7)*	Yes	1e
rs368220166	K332N	1	−0.42	Decrease (2)	Yes	2e
rs371728648	G346S	5	−0.27	Decrease (7)	No	8b
**rs191847788**	**K364N**	**4.5**	**−1.09**	**Decrease (4)**	**No**	**9e**
**rs375595000**	**P403T**	**6**	**−2.64**	**Decrease (8)**	**No**	**8e**
rs370495523	L432W	4	0.08	Decrease (6)	No	8b
rs187416296	R442C	5	−1.23	Decrease (6)*	No	7e
rs377529439	F456I	5	−1.59	Decrease (8)*	No	8b (9b)
*rs371028900*	*T460I*	*6*	*−1.38*	Decrease (5)*	*Yes*	*9b*
rs200638791	P484S	4.5	−2.97	Decrease (9)*	No	6e (9b)
rs200148337	C494F	6	−0.21	Decrease (4)	No	8b

# Del. Pred.  =  number of deleterious predictions; nsSNPs with 4 or more deleterious predictions are considered high-risk nsSNPs, while nsSNPs with less than 4 deleterious predictions are considered low-risk; DDG: free energy change value in Kcal/mol (>0 increase, <0 decrease, >0.5 large increase, <−0.5 large decrease); Sign of DDG: the direction of the change (increase or decrease); The reliability index (RI) from 0–9 is shown in parentheses, where 0 is the lowest RI and 9 is the highest); PTM: predicted post-translational modification site; ConSurf results are shown in the last column (number represents the conservation score (CS) from 1–9, letter represents whether the residue was predicted to be exposed (e) or buried (b), putative functional residues are indicated with bold text; whereas putative structural residues are indicated with italicized text (Figure S1); Sites with an additional ConSurf result in parentheses are located next to putative functional (9e) or structural (9b) residues; nsSNPs with the largest predicted stability decreases (DDG <−1.0) that also have a RI score of ≥5 are indicated with an asterisk.

**Figure 3:**
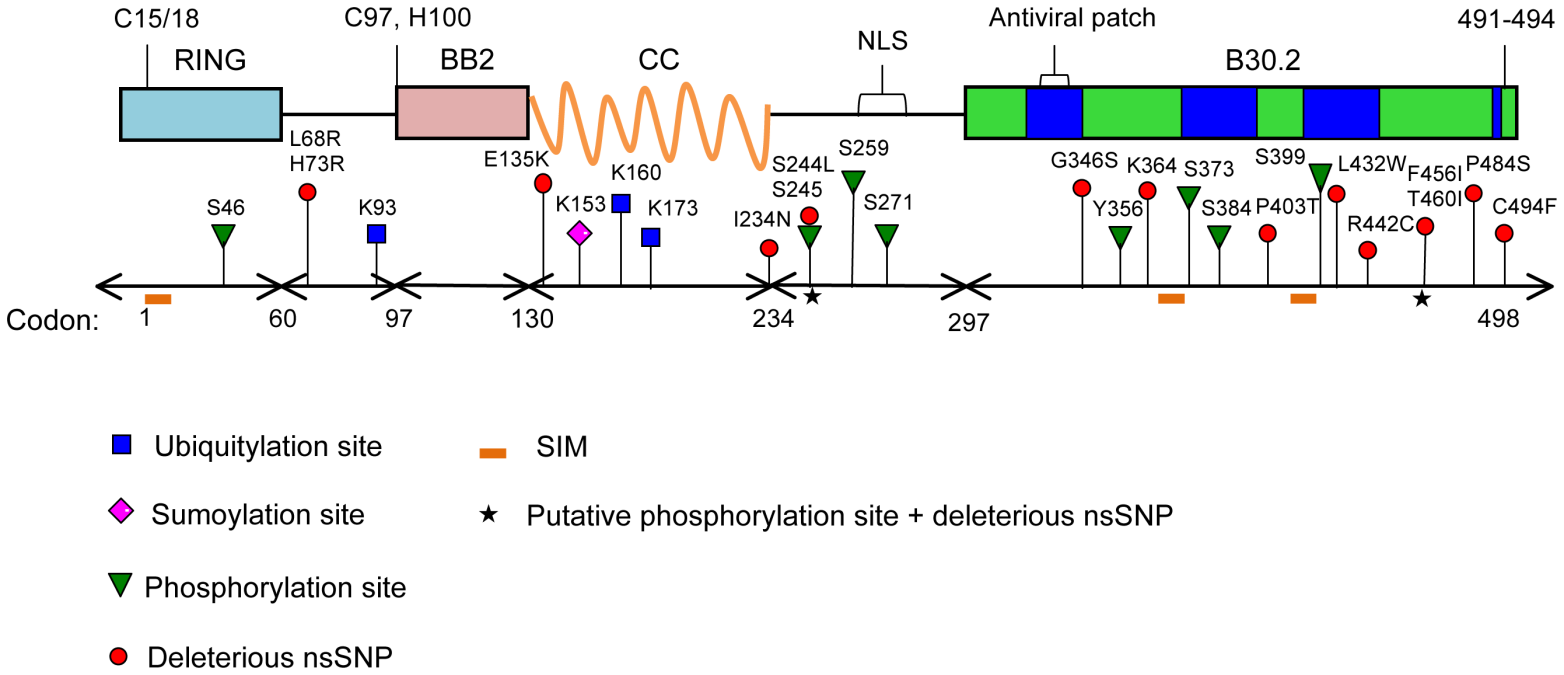
Putative functional sites in the TRIM22 protein. Schematic depicting the approximate location of the top predicted PTM sites (ubiquitylation, sumoylation, and phosphorylation), the 14 high-risk nsSNPs in TRIM22, the 3 sumo-interacting motifs (SIMs), and the 2 high-risk nsSNP sites (S244L and T460I) predicted to undergo phosphorylation in the wild type TRIM22 protein. Several sites of known functional importance are marked on the TRIM22 protein (top image), including the C15/C18 residues (required for TRIM22 E3 ligase activity), the C97/H100 residues (part of the zinc-binding motif in BB2), and the nuclear localization signal (NLS) [81]–[83]. The ‘antiviral patch’ region, which was previously shown to be integral for the antiviral activity of TRIM5α, is shown in the B30.2 domain, as well as the approximate location of each variable region (v1-v4, bright blue areas) [28], [33]. Amino acids 491–494 were previously shown to be required for the nuclear localization of TRIM22 [84]. RING, B-box 2 (BB2), coiled-coil (CC), and B30.2 (PRY/SPRY) domains are listed.

It is possible that the phosphorylation of TRIM22 at sites S244 and/or T460 is required for some integral TRIM22 function and that the nsSNPs S244L and T460I impair this function; however, these nsSNPs may also impair protein stability, which would likely amplify any detrimental of PTM impairment. Many additional high-risk nsSNPs, plus several low-risk nsSNPs located at putative PTM sites, also decreased TRIM22 protein stability (Table 7). A number of studies have shown that decreased protein stability leads to increased protein misfolding, aggregation, and degradation. Accordingly, decreased stability typically results in decreased net function [77]–[80]. Future in-depth studies are required to investigate the effects of these nsSNPs on the structure and function of TRIM22's B30.2 domain. Pertinent TRIM22 sites that are predicted to be highly deleterious and/or undergo PTMs are depicted in Figure 3.

## Conclusions

Our results demonstrate that multiple nsSNPs in the antiviral *TRIM22* gene may be deleterious to TRIM22 structure and/or function. Most of these high-risk nsSNPs are located at highly conserved amino acid sites in a protein-protein interaction module called the B30.2 domain. In this study, we show that 9 of the top high-risk nsSNPs disrupt the putative structure of TRIM22's B30.2 domain, particularly the surface-exposed v2 and v3 regions. In the closely-related TRIM5α protein, these same regions were previously shown to play a key role in retroviral restriction. In addition to these findings, we also identify several TRIM22 sites that may undergo post-translational modification, including sites that coincide with the location of high-risk nsSNPs. This study is the first systematic and extensive *in silico* analysis of functional SNPs in the *TRIM22* gene.

## Supporting Information

Figure S1 **ConSurf analysis of amino acid sites in the TRIM22 protein.** Schematic showing ConSurf results for the human TRIM22 protein. Amino acids were ranked on a conservation scale of 1–9 and are highlighted as follows: blue residues (1–4) are variable, white residues (5) are average, and purple residues (6–9) are conserved. Residues predicted to be exposed to the surface of the protein are indicated via an orange letter ‘e’, while residues predicted to be buried are indicated via a green letter ‘b’. Putative structural residues are demarcated with a blue letter ‘s’ (highly conserved and buried), whereas putative functional residues are demarcated with a red letter ‘f’ (highly conserved and exposed).
(PDF)

Figure S2 **ConSurf analysis of amino acid sites in a variety of aligned primate TRIM22 protein sequences.**
(PDF)

Table S1 **Non-synonymous SNPs in the TRIM22 protein.**
(XLSX)

Table S2 **Prediction results for nsSNPs in the TRIM22 protein.**
(XLSX)
