# Supplementary figures and images for: In Silico Analysis of Functional Single Nucleotide Polymorphisms in the Human TRIM22 Gene
Source: PLoS One. 2014 Jul 1;9(7):e101436. doi: 10.1371/journal.pone.0101436 (PMC4077803; doi:10.1371/journal.pone.0101436)

# ConSurf Results

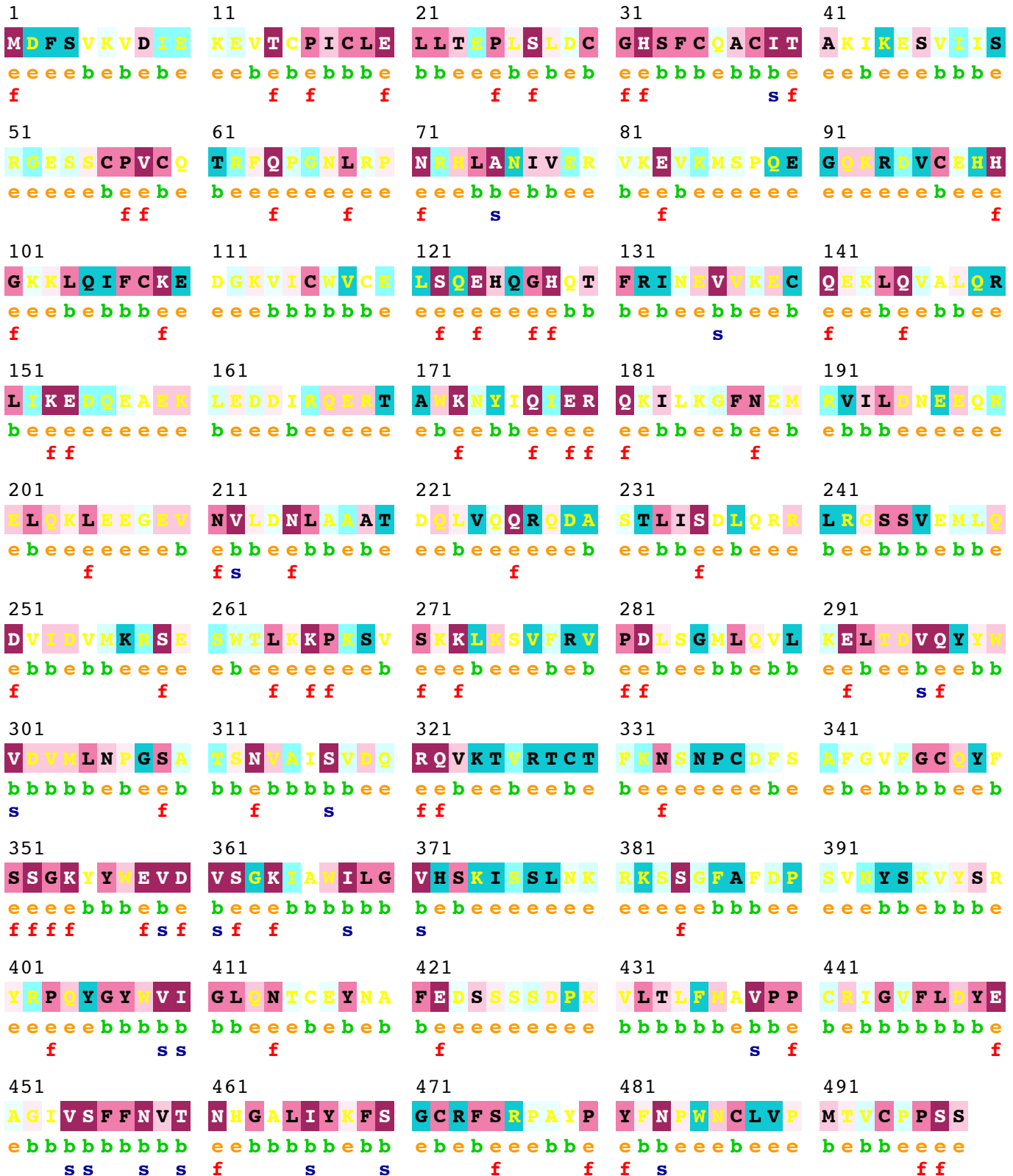

The conservation scale:

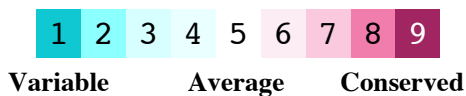

Supplement: Figure S1 — ConSurf analysis of amino acid sites in the TRIM22 protein. Schematic showing ConSurf results for the human TRIM22 protein. Amino acids were ranked on a conservation scale of 1–9 and are highlighted as follows: blue residues (1–4) are variable, white residues (5) are average, and purple residues (6–9) are conserved. Residues predicted to be exposed to the surface of the protein are indicated via an orange letter ‘e’, while residues predicted to be buried are indicated via a green letter ‘b’. Putative structural residues are demarcated with a blue letter ‘s’ (highly conserved and buried), whereas putative functional residues are demarcated with a red letter ‘f’ (highly conserved and exposed). (PDF) [file pone.0101436.s001.pdf]
