# Supplementary material for: In Silico Analysis of Functional Single Nucleotide Polymorphisms in the Human TRIM22 Gene
Source: PLoS One. 2014 Jul 1;9(7):e101436. doi: 10.1371/journal.pone.0101436 (PMC4077803; doi:10.1371/journal.pone.0101436)
Supplement: Figure S2 — ConSurf analysis of amino acid sites in a variety of aligned primate TRIM22 protein sequences. (PDF) [file pone.0101436.s002.pdf]

ConSurf Color-Coded MSA

```
Human           MDFSVKVDIEKEVTCPICLELLTEPLSLDCGHSFCQACITAKIKE--SVI
Panda           MDFPAQVNIQKDVTCPICLELLTMPLSLDCGHSFCQACITAKSKE--SGT
Baboon          MDFSVKVDIEKEVTCPICLELLTEPLSLDCGHSFCQACITAKIKE--SVT
Gibbon          MDFSVKVDIEKEVTCPICLELLTEPLSLDCGHSFCQACITAKIKE--SVI
SquirrelMonkey  MELSVKVDIGKEVTCPICLELLTDPLSIDCGHSFCHACITAKNK---SVI
Rhino           MDLQAQVNLQKEVTCPICLELLTEPLSLGCGHSFCKACITAKNKE--SVI
Dog             MDFPAQANIQKDVTCPICLELLTMPLSLDCGHSFCQACITAKSME--SGT
Cat             MDFLAQVNLKRELTYPICLELLTEPLSLXCGHTFCXDCITAKNRD----S
Manatee         -MASVQANIEKEVTCPICLELLTKPLSLNCGHSFCQACITAKNVELQTVI
RhesusMacaque   MDFSVKVDIEKEVTCPICLELLTEPLSLDCGHSFCQACITAKIKE--SVT
Elephant        MASKILVNLEEGATCPICLELLMEPISLDCGHSFCQACITADNKK--SMV
Orangutan       MDFSVKVDIEKEVTCPICLELLTEPLSLDCGHSFCQACITAKIKE--SVI
Gorilla         MDFSVKVDIEKEVTCPLCLELLTEPLSLDCGHSFCQACITAKIKE--SVI
Chimpanzee      MDFSVKVDIEKEVTCPICLELLTEPLSLDCGHSFCQACITTKIKE--SVI
```

```
Human           ISRGESSCPVCQTRFQPGNLRPNRHLANIVERVKEVKMSPQEGQKRDVCE
Panda           HKGGESNCPVCQCKYQFWNLRPNQPLANIVKKVRENM----SPQQKKLCK
Baboon          ISRGESSCPVCQSRFQPGKLRPNRHLANIVERVKEVKMSPQEGQKRDICE
Gibbon          ISRGESSCPVCQTRFQPGNLRPNRHLANIVERVKEVKMSPQEGQKRDVCE
SquirrelMonkey  ISGGQSSCPVCQTRFQPGNLRPNRHLANIVQRVREVKMGPEDGQKRDVCE
Rhino           HPGGECSCPVCQSRYQPGTLQPNRHLATIVERVREINTNPRKQQKKEFCE
Dog             HQGEEGTCPVCQCKYQFWNLRPNQPLANIAKKVRENM----SPQKQKLCE
Cat             XQGGESNCPVCQCRYQLQNLXPNHPLAGIVERVRENR----SPQKKKFCE
Manatee         SPGGQKSCPVCHASYQPGNLWPNQHLAKIVKGFREAKLSPQEGQKSDRCV
RhesusMacaque   ISRGESSCPVCQSRFQPGKLRPNRHLANIVERVKEVKMSPQEGQKRDICE
Elephant        SQEKESSCPVCRIKYQPGNLRSNQHLASMVERLKEVKMSLEKEEKENYCV
Orangutan       ISRGESSCPVCQTRFQPGNLRPNRHLANIVERVKEVKMSPQEGQKRDVCE
Gorilla         ISRGESSCPVCQTRFQPGNLRPNRHLANIVERVKEVKMSPQEGQKRDVCE
Chimpanzee      ISRGESSCPVCQTRFQPGNLRPNRHLANIVERVKEVKMSPQEGQKRDVCE
```

```
Human           HHGKKLQIFCKEDGKVICWVCELSQEHQGHQTFRINEVVKECQEKLQVAL
Panda           HHGEKLVIFCKEDGKAICQRCAQSVEHHGHQIFFMEKVIKECQEKLQAAL
Baboon          HHGKKLQIFCKEDGKVICWVCELSQEHQGHQTFRINEVVKECQEKLQAAL
Gibbon          QHGKKLQIFCKEDGKVICWVCELSQEHQGHQTFRINEVVKECQEKLQAAL
SquirrelMonkey  HHGKKLQYFCKEDGKVICWVCELSQEHQGHQTFLIHEVVKECQERLQAAL
Rhino           HHGEKLHMFCKDEEQAICRLCVLSREHQGHQIFCMEEVVMKIQEKLQEAL
Dog             VHGEKLLTFCKEDGKAICQHCAQSAEHRGHQIFFIEKVIKEYQEKLQAAL
Cat             NHGKTLGVFCKEHGKAGCQHTAVSQEYHGHHIFLTEKVVKECQEKLQATL
Manatee         HHGEKLLLFCKNDEKVICWLCVRSQEHCGHQTFLVEEVVKECQEKLQETL
RhesusMacaque   HHGKKLQIFCKEDGKVICWVCELSQEHQGHQTFRINEVVKECQEKLQAAL
Elephant        HHGEKLRLFCKLDGKFICWLCERSQEHHGHPTFLVEEVVPEYQKNLQAAF
Orangutan       HHGKKLQIFCKEDGKVICWVCELSQEHQGHQTFRINEVVKECQEKLQVAL
Gorilla         HHGKKLQIFCKEDGKVICWVCELSQEHQGHQTFRINEVVKECQEKLQVAL
Chimpanzee      PHGKKLQIFCKEDGKVICWVCELSPEHQGHQTFRINEVVKECQEKLQVAL
```

```
Human           QRLIKEDQEAEKLEDDIRQERTAWKNYIQIERQKILKGFNEMRVILDNEE
Panda           KKLRKEEPKMEEWEADIREEKASWKNHMQTERQRILKGFNEMKAILDSEE
Baboon          QKLTKEDQEAEKLEDDIRQERTIWKNYIQIERQKILKGFNEMRVILDSEE
Gibbon          QKLIKEDQEAEKLEDDIRQERTTWKNHIQIERQKILKGFNEMRVILDSEE
SquirrelMonkey  QRLIKEDQEAEKLEVDIQQERTSWKSYIQTERQKILKGFNEMRVILDSEE
Rhino           KKLRKEQQEAAELEADIREERATWKNHMQTERQRILKGFNEMRCILDSLE
Dog             KKLRKEHTKVEELEADIKEKRVSWKNHMQSERQRILKGFNEMRDILNSEE
Cat             KKLRKEQQKVEELEVDIREERDSWKNHMQTERQRILKEFNEMKGLLDSKE
Manatee         TRLRKEQQEAEKLEAQIIEERASWKHQTQTERQRILAGFNDLHAILDNDK
RhesusMacaque   QKLTKEDQEAEKLEDDVRQERTVWKNYIQLERQKILKGFNEMRVILDSEE
Elephant        ESLSKEQKKAEKWKADLREERDSWKKYIQTERQSVRENFSQLRSILDNEE
Orangutan       QRLIKEDQEAEKLEDDIRQERTTWKNYIQIERQKILKGFNEMRVILDNEE
Gorilla         QRLIKEDQEAEKLEDDIRQERTAWKNYIQKERQKILKGFNEMRVILDNEE
```

```
Chimpanzee        QRLIKEDQEAEKLEDDIRQERTAWKNYIQIERQKILKGFNEMRVILDNEE

Human             QRELQKLEEGEVNVLDNLAAATDQLVQQRQDASTLISDLQRRLRGSSVEM
Panda             KRVLQKLEEDEVNVLDNLVVAKDQLARQKQCLRELISAVEHQIWGSSVDT
Baboon            QRELQKLEEGEVNVLDNLAAASDQLVQQRQDASKLISDLQRRLRGSSIEM
Gibbon            QRELQKLEEGEVNVLDNLAAATDQLVQQRQDASKLISDLQRRLRGSSVEM
SquirrelMonkey    QRELQKLEEGEVNVLDNLAEAKDQLVQQRQYASKLISDLQRRIKGSSVEM
Rhino             QKELQKLEEDEVNVLENLVAAKDQLVQQSQYMRELISDLERQMWGSSIDT
Dog               KKVLQKLEEDEVNVLDNLIVARDQFAQQKQCLRELISDIEHQIWGSSVDT
Cat               KRVLQKLEEDKVNVLDNLVVARDQLDWQRQYLRGLISHIEHQIWGSSEDM
Manatee           KRELQKLEEEEVNVLDNLAEVKYQLIQQYQDLSDLISDVEHRMQWSSVDT
RhesusMacaque     QRELQKLEEGEVNVLDNLAAARDQLVQQRQDASKLISDLQRRLRGSSIEM
Elephant          QKELEELKSEEESVMQNLSEAENELGQQSDDVKKLMSDLQHRLQGTTMEM
Orangutan         QRELQKLEEGEVNVLDNLAAATDQLVQQRQDASTLISDLQRRLRGSSVEM
Gorilla           QRELQKLEEGEVNVLDNLAAATDQLVQQRQDASTLISDLQRRLRGSSVEM
Chimpanzee        QRELQKLEEGEVNVLDNLAAATDQLVQQRQDASTLISDLQRRLRGSSVEM

Human             LQDVIDVMKRSESWTLKKPKSVSKKLKSVFRVPDLSGMLQVLKELTDVQY
Panda             VQDMINVMKRSESWTLKKPNTASKKLKSTFRVPDLSGMLQVFKELTEVQR
Baboon            LQDVIDVMKRSENWTLKKPKPVSKKLKSVFRAPDLSRMLQVHKELTDVQC
Gibbon            LQDVIDVMKRSESWTLKKPKSVSKKLKSVFRVPDLSGMLQVLKELTDVQY
SquirrelMonkey    LQDVIDVMRSSETWILKKPKSVSKKLKSIFQMPDLSGMLQILRELTDVQC
Rhino             LRDVINLIRRSETWTLKKPKMVSKKQKSAFRVPDLSGMLQVFKELTEVQC
Dog               VQDMINVMKRSESWTLKKPNIVSKKLKSTFLIPDLSGMLQVFKELTEVQC
Cat               LQDLINVMRKSESWILKKPHIVSKKSKSTYQIPDLKGTLQMFKELTEVQR
Manatee           MQDVIDIVKRSESWTLKKPTIVSKKLKNVCRVPDLKEILQVFRELTDVQC
RhesusMacaque     LQDVIDVMKRSENWTLKKPKPVSKKLKSVFRAPDLSRMLQVHKELTDVQC
Elephant          LQDVNDVIQGSKNTILEKPKTLPQKPRRIFQVPDMSLILCVMRELEDVQR
Orangutan         LQDVIDVMKRSESWTLKKPKSVSKKLKSVFRVPDLSGMLQVLKELTDVQY
Gorilla           LQDVIDVMKRSESWTLKKPKSVSKKLKSVFRVPDLSGMLQVLKELTDVQY
Chimpanzee        LQDVIDVMKRSESWTLKKPKSVSKKLKSVFRVPDLSGMLQVLKELTDVQY

Human             YWVDVMLNPGSATSNVAISVDQRQVKTVRTCTFKNSN-PCDFSAFGVFGC
Panda             YWVDVMLKPVNAISNIAISADKRQVTAVHNLSLKNIF-LCDFSAFDILGC
Baboon            HWVDVMLNPNSTISNVAVSVDQRQVATVRTFAFKNSN-PRDFSDFGVLGC
Gibbon            YWVDVMLNPVSATSNVAVSADQRQVKTVRTCTFKNSN-PCDFSAFGVFGC
SquirrelMonkey    YWVDVMLNPDSATLNVAVSADQRQVATVHTSTFKNSN-PRDFSAFGVLGC
Rhino             YWVDVMLNPVNAISNIVISADQRQVKILHPFTFRNVY-PCDFSAFDVLGC
Dog               YWVDVMLKPVNAISNITISADKRQVTVVHNFPLKSIY-PFDFSAFDILGC
Cat               YWVDVMLKPVNAILNITISADKRQVKGIQDFSLQNIH-LCSFPLFDVLGC
Manatee           YWVDVTLNQVNTSSNVVISADQRQVAVLRIPNFRNSD-QHDFSAFDVLGC
RhesusMacaque     YWVDVMLNPNSTISNVAVSVDQRQVATVRTFTFKNSN-PRDFSDFGVLGC
Elephant          YWVNMTLNPINPISNVVISEGGRQARIVPNSKSRNFCWSGSNDDDGIVGC
Orangutan         YWVDVMLNPGSATSNVAVSVDQRQVKTVRTCTFKNSN-PCDFSAFGVFGC
Gorilla           YWVDVMLNPGSATSNVAISVDQRQVKTVHTCTFKNSN-PCDFSAFGVFGC
Chimpanzee        YWVDVMLNPGSATSNVAISVDQRQVKTVRTCTFKNSN-PCDFSAFGVFGC

Human             QYFSSGKYYWEVDVSGKIAWILGVHSKISSLNKRKSSGFAFDPSVNYSKV
Panda             QHFSSGKYYWEVDVSEKTAWILGVYSKARNLKRKGSSGFVFDPNVNHPDV
Baboon            QHFSSGKYYWEVDVSGKIAWILGVYSKISSPNKRKSSGFVFDPSVNYANV
Gibbon            QYFSSGKYYWEVDVSGKIAWILGVHSKISSLNKRKSSGFAFDPSVNYSKV
SquirrelMonkey    QHFSSGKYYWEVDVSGKSAWILGVHSKISNPNKRKSSGFTFDTSTNYSSI
Rhino             QCFSSGKYYWEVDVSGKIAWILGVCSNKRCFNRTKSSGFTFDPNLNHSNV
Dog               QHFSSGKYYWEVDVSGKIAWILGVYSKGRSLKRKGDSGFFFDPKVNHLDV
Cat               QHFSXGKYYWEVDVSEKISWILGVNSKARSPKRKGSSGFVFDPNVYLPDV
Manatee           QYFSSGKHYWEVDVSRKTAWILGVYCRAKDPHERWASVFAFGLNVSSQNV
RhesusMacaque     QYFSSGKYYWEVDVSGKIAWILGVYSKISSPNKRKSSGFVFDPSVNYANV
Elephant          LYIASGKHYWEVDVSGKTSWILGVCSDRCPE----FSMLGAKQGVYLQND
Orangutan         QYFSSGKYYWEVDVSGKIAWILGVHSKISSLNKRKSSGLAFDPSVNYSKV
```

```
Gorilla          QYFSSGKYYWEVDVSGKIAWILGVHSKISSLNKRKSSGLAFDPSVNYSKV
Chimpanzee       QYFSSGKYYWEVDVSGKIAWILGVHSKISSLNKRKSSGFAFDPSVNYSKV


Human            YSRYRPQYGYWVIGLQNTCEYNAFEDSSSSDPKVLTLFMAVPPCRIGVFL
Panda            YSRYKPQYGYWVIGLQNESEYKVFEESSTSDPKVLTLFMPVPPCRIGTFL
Baboon           YSKYRPQYGYWVIGLQNTCEYNAFEDSSSADPKVLTLFMAVPPCRVGVFL
Gibbon           YSKYRPQYGYWVIGLQNTCEYNAFEDSSSSDPKVLTLFMAVPPCRIGVFL
SquirrelMonkey   YSKYRPQCGYWVIGLQDTSEYNAFEDSSSSDLKVLTLFMAVPPCRIGIFL
Rhino            YSRYRPQYGYWVIGLQNESEYKAFEDSSTSDPKVLTLSMAVPPRRVGVFL
Dog              YSRFKPQLGYWVIGLQNESEYKVFEESSTSDSKVLTLFMPVPPCRIGVFL
Cat              YSRYNPQFGYWVIGLQNESGYKVFEESSTFDPKVLTLCMTVPP-HIGVFL
Manatee          FSRYRPQYGYWVIGLQNQFEYNAFENSSTSNPQVLTLFMAVPPRRIGVFL
RhesusMacaque    YSKYRPQYGYWVIGLQNTCEYNAFEDSSSADPKVLTLFMAVPPCRVGVFL
Elephant         YFRYQPKYGYWVIGLENQSGYKVFENLCLG-SQNLIFSLAVPPCRVGIFL
Orangutan        YSKYRPQYGYWVIGLQNTCEYNAFEDSSSSDPKVLTLFMAVPPCRIGVFL
Gorilla          YSKYRPQYGYWVIGLQNTCEYNAFEDSFSSDPKVLTLFMAVPPCRIGVFL
Chimpanzee       YSKYRPQYGYWVIGLQNTCEYNAFEDSSSSDPKVLTLFMAVPPCRIGVFL


Human            DYEAGIVSFFNVTNHGALIYKFSGCRFSRPAYPYFNPWNCLVPMTVCPPS
Panda            DYEARIVSFFNVTNHGSLIYKFSNCQFSQTTYPYFNLWNCQRPITVCPPS
Baboon           NYEAGIVSFFNVTNHGALIYKFSGCRFSQPAYPYFNPWNCQVPMTLCQPS
Gibbon           DYEAGIVSFFNVTNHGALIYKFSGCRFSQPAYPYFNPWNCLVPMTVCPPS
SquirrelMonkey   DYEAGIVSFFNVTNHGALIYKFSRCRFSQPAYPYFNPWNCPAPMTLCPPS
Rhino            DYEGGTVSFFNVTNHGSLIYKFSKCHFSQTAYPYINPWNCPAPMTLCPPS
Dog              DYEARIVSFFNVTNHGSLIYKFSECQFSQTTYPYFNPWNCQYPITLCPPS
Cat              DYEAGIVSFFNITNHGSLIYEFSRCQFFQTTYPYFNPWNCPGSIILCPPS
Manatee          DYEAGTVSFFNVTNQGSLIYKFSQCCFSQCVCPYFNPCKCPAPMTLCPPS
RhesusMacaque    NYEAGIVSFFNVTNHGALIYKFSGCRFSQPAYPYFNPWNCQVPMTLCQPS
Elephant         DYEFGTVSFFNVTNYGFLIYKFSNCCFSGTVYPYFNPMKCEVPMSLCLPS
Orangutan        DYEAGIVSFFNVTNHGALIYKFSGCRFSRPAYPYFNPWNCLVPMTVCPPS
Gorilla          DYEAGIVSFFNVTNHGALIYKFSGCRFSQPAYPYFNPWNCLVPMTVCPPS
Chimpanzee       DYEAGIVSFFNVTNHGALIYKFSGCHFSRPAYPYFNPWNCLVPMTVCPPS


Human            S-----------------------------
Panda            S-----------------------------
Baboon           S-----------------------------
Gibbon           S-----------------------------
SquirrelMonkey   S-----------------------------
Rhino            S-----------------------------
Dog              S-----------------------------
Cat              SGTFLAPPHFCVVLLVLGAHLLHLSSSAPF
Manatee          S-----------------------------
RhesusMacaque    S-----------------------------
Elephant         F-----------------------------
Orangutan        S-----------------------------
Gorilla          S-----------------------------
Chimpanzee       S-----------------------------
```

1	2	3	4	5	6	7	8	9

Variable Average Conserved

X - Insufficient data - the calculation for this site was performed on less than 10% of the sequences.
